# Binding of Hydrophobic Guests in a Coordination Cage Cavity is Driven by Liberation of “High‐Energy” Water

**DOI:** 10.1002/chem.201704163

**Published:** 2017-11-30

**Authors:** Alexander J. Metherell, William Cullen, Nicholas H. Williams, Michael D. Ward

**Affiliations:** ^1^ Department of Chemistry University of Sheffield Sheffield S3 7HF UK; ^2^ Department of Chemistry University of Warwick Coventry CV4 7AL UK

**Keywords:** coordination cage, host–guest systems, hydrophobic effect, molecular recognition, supramolecular chemistry

## Abstract

The cavity of an M_8_L_12_ cubic coordination cage can accommodate a cluster of ten water molecules in which the average number of hydrogen bonds per water molecule is 0.5 H‐bonds less than it would be in the bulk solution. The presence of these “hydrogen‐bond frustrated” or “high‐energy” water molecules in the cavity results in the hydrophobic effect associated with guest binding being predominantly enthalpy‐based, as these water molecules can improve their hydrogen‐bonding environment on release. This contrasts with the classical form of the hydrophobic effect in which the favourable entropy change associated with release of ordered molecules from hydrophobic surfaces dominates. For several guests Van't Hoff plots showed that the free energy of binding in water is primarily enthalpy driven. For five homologous pairs of guests related by the presence or absence of a CH_2_ group, the incremental changes to Δ*H* and *T*Δ*S* for guest binding—that is, ΔΔ*H* and *T*ΔΔ*S*, the difference in contributions arising from the CH_2_ group—are consistently 5(±1) kJ mol^−1^ for ΔΔ*H* and 0(±1) kJ mol^−1^ for *T*ΔΔ*S*. This systematic dominance of Δ*H* in the binding of hydrophobic guests is consistent with the view that guest binding is dominated by release of “high energy” water molecules into a more favourable solvation environment, as has been demonstrated recently for some members of the cucurbituril family.

## Introduction

Of the factors that control host‐guest binding in water, whether using biological or artificial receptors, the hydrophobic effect is probably the most important and yet is still poorly understood.^1^, ^2^, ^3^, ^4^ The favourable free energy change associated with bringing together hydrophobic surfaces of host and guest species that become desolvated was originally explained in terms of a favourable entropy change arising from the liberation of ordered water molecules at the interfaces.^2^ However it has become apparent that this is not always true as some systems show large favourable enthalpic contributions to the hydrophobic effect, with the balance between enthalpy and entropy contributions being strongly dependent on the particular system.^1^, ^3^ Whether Δ*H* or Δ*S* dominates the hydrophobic effect depends on details of the local structure of water around the particular hydrophobic surface, to the extent that convex, flat, and concave surfaces of the same surface area can provide widely different Δ*H* or Δ*S* contributions to guest binding.^3^

In particular, concave surfaces whose shape limits the space around water molecules that are in contact with them, thereby limiting the ability of the water molecules to find hydrogen‐bonding partners, can result in “high‐energy” water molecules that are likely to gain enthalpic stabilisation from being liberated into the bulk solvent where the hydrogen‐bonding environment is unconstrained.^4^ This is a situation that applies particularly to the cavities in synthetic hosts. Recently it has been shown that a high favourable Δ*H* contribution to guest binding occurs in synthetic hosts such as cucurbiturils.^4^, ^5^, ^6^, ^7^ The arrangement of water molecules in these small cavities is such that each one forms, on average, fewer hydrogen bonds than it would in bulk solution and is thus energetically “frustrated”. The degree of frustration per water molecule (i.e. the deficiency in the average number of hydrogen bonds formed, compared to what can happen in bulk solution), multiplied by the number of water molecules liberated from the cavity when a guest binds, gives a rough estimate of the enthalpic stabilisation associated with the hydrophobic contribution to guest binding. This combination explains why the cucurbituril heptamer (CB7) provides remarkably strong binding of hydrophobic guests that is unmatched by any other synthetic host and makes it stand out from its smaller and larger analogues CB6 and CB8.^5^ Whilst the water molecules in CB6 are individually more frustrated than those in CB7, there are far fewer of them. Conversely, there are more water molecules in the cavity of CB8 that can be displaced by a guest, but each one has more hydrogen‐bonding partners when in the cavity, so the enthalpic frustration of each is reduced. For CB7 the trade off between the number of water molecule guests, and the degree of hydrogen‐bond frustration of each, is such that the favourable enthalpy change on release of the water molecules when a guest binds substantially dwarfs the entropy contribution associated with liberating bound solvent molecules.^5^ Clearly this principle is of great importance in allowing supramolecular chemists to design optimal synthetic hosts: if the structural criteria for optimising the amount of “energetically frustrated” or “high‐energy” water in a cavity can be combined with shape/size complementarity for guests, this will improve the design of new synthetic receptors.^4^, ^5^

**Figure 1:**
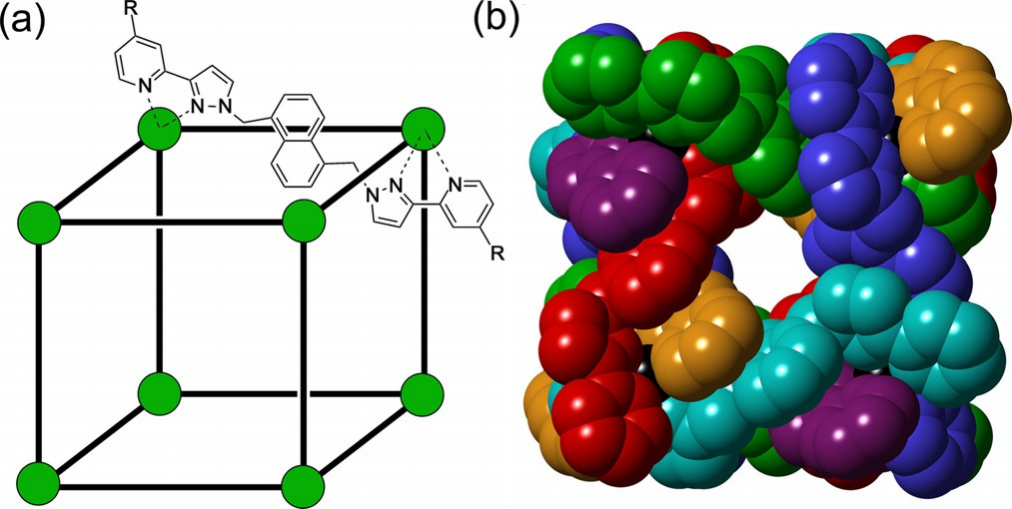
The octanuclear [M_8_L_12_](BF_4_)_16_ cages used in this work (**H**, R=H, ref. 10a; **H^w^** ^,^ R=CH_2_OH, ref. 10b). a) A sketch showing the approximate arrangement of metal ions and the structural formula of the bridging ligands, which span every edge of the cube; b) a view of the cationic cage cavity with each ligand coloured separately (from ref. 10a).

Whilst there are many synthetic capsules whose guest binding properties have been investigated, based on either organic hydrogen‐bonded or halogen‐bonded assemblies^8^ or metal/ligand coordination cages,^9^ the number of those that have a well‐developed, quantitative understanding of the factors underpinning guest binding in water is very limited. The octanuclear, approximately cubic, M_8_L_12_ coordination cages9l, 10 shown in Figure 1 (**H** is the parent cage that is water‐insoluble;10a **H^w^** is substituted on the exterior surface with hydroxymethyl groups to aid water solubility10b) constitute a host system in which the factors responsible for guest binding in different solvents have been studied in considerable detail. In particular the contributions of hydrogen‐bonding of polar guests to the interior surface,^11^ the magnitude of hydrophobic contributions to binding as a function of guest surface area,10b, 12 and the effects of conformational entropy on guest binding strengths,^13^ have all been explicitly analysed. The culmination of this is the development of a scoring function which allows the in silico prediction of guest binding constants in the cage cavity, using the molecular docking programme GOLD, with high reliability.^13^, ^14^ Accordingly this cage provides an ideal system in which to probe in detail the contributions to binding of hydrophobic guests.

We report here a combined crystallographic and NMR spectroscopic study which reveals that the strong binding of guests in the cage cavity in water has a substantial enthalpy component, and propose that this arises from the energetic frustration of cage‐bound water in the free cage—so‐called “high‐energy water”, as in the CB series of hosts.^4^, ^5^ Using an incremental approach, by comparing pairs of similar guests that differ by just a methylene (CH_2_) group, we show how we can quantify the additional Δ*H* and Δ*S* contributions to the hydrophobic effect of guest binding arising from the extra CH_2_ units.

## Results and Discussion

### Structure of the hydrated host cage

**Figure 2:**
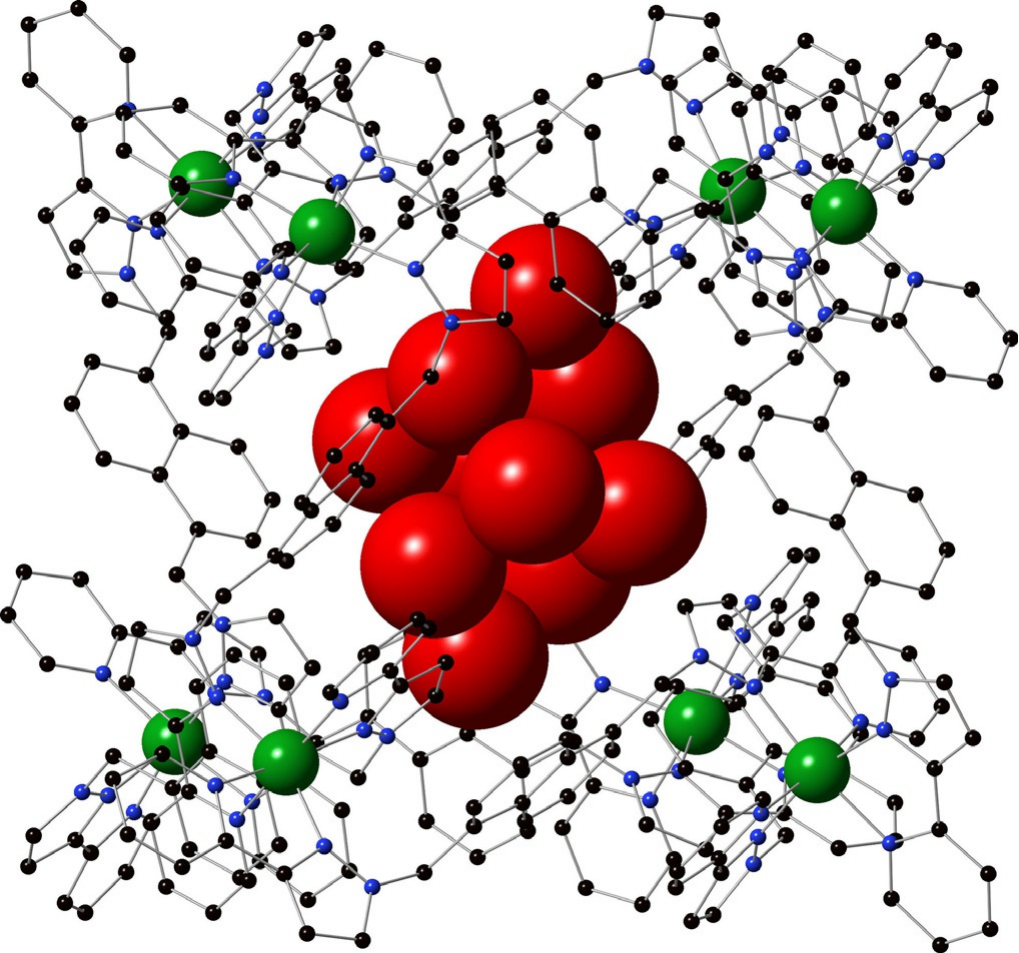
Crystal structure of the complex cation of **H**⋅(H_2_O)_28_ showing the ten water molecules (major disorder component) in the central cavity. For additional figures see the Supporting Information.

We have found in earlier work^12^, ^13^, ^15^ that pre‐formed crystals of the host **H**, [Co_8_L_12_](BF_4_)_16_, can take up guests into the cavity by soaking the crystals either in the pure guest (if it is an oil or a liquid) or a solution of the guest (if it is a solid)—the Fujita “crystalline sponge” method.^16^ Placing crystals of **H** 10a in water for a few hours and then determining the crystal structure by X‐ray diffraction showed the structure to be **H**⋅(H_2_O)_28_ in which the cage cavity is now occupied by a cluster of ten water molecules. The cage has twofold symmetry so the asymmetric unit contains half of the cage and five water molecule guests, all five being disordered over two closely‐spaced positions (see the Supporting Information). A view of the cage in wireframe, with its collection of guest water molecules shown space‐filling (major disorder component only) is shown in Figure 2.

**Figure 3:**
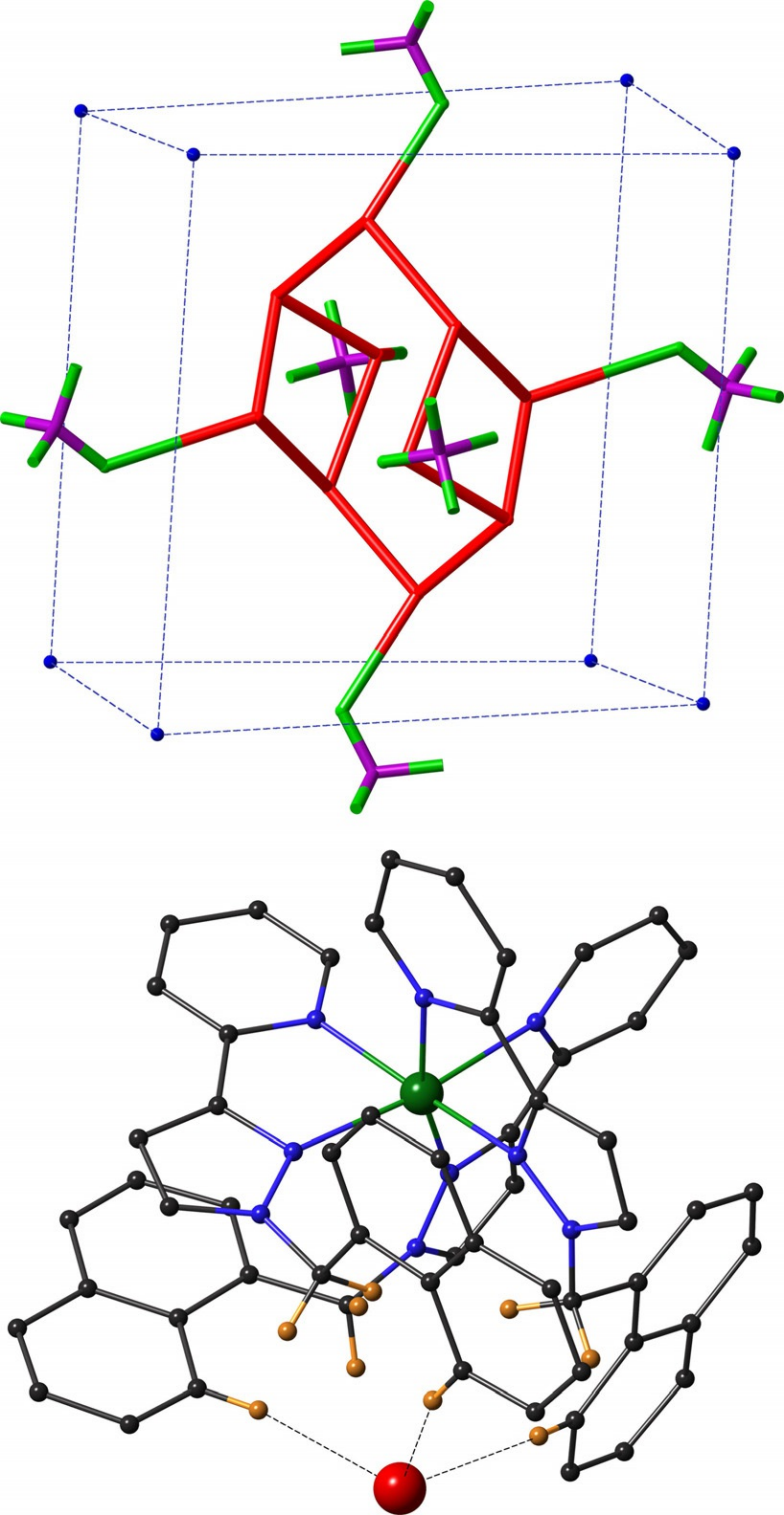
Two additional views of the crystal structure of **H**⋅(H_2_O)_28._ a) The ten bound water molecules (major disorder component) and their interactions with the peripheral fluoroborate anions that occupy the windows in the cube faces; solid lines denote O⋅⋅⋅O or O⋅⋅⋅F contacts of ≤3.2 Å. b) Interaction of one bound water molecule with the hydrogen‐bond donor pocket on the interior surface of the cage at a *fac* tris‐chelate vertex (there is a symmetrically equivalent interaction at the diagonally opposite vertex). Dotted lines indicate CH⋅⋅⋅O contacts of <3 Å; the O(1G)⋅⋅⋅Co(1) distance is 5.82 Å.

Closer views of the water molecules, as well as the six tetrafluoroborate anions that lie around the cage surface occupying the portals, are shown in Figure 3 (only the major disorder component is shown). The bound water molecules do not adopt a perfectly close‐packed “ice‐like” arrangement as has been seen in some other cases,^17^ which is presumably partly due to the constraints of the cavity shape. We note that the structures of water clusters have been of significant interest recently and many types of assembly have been identified.^18^ In **H**⋅(H_2_O)_28_ six of the cavity‐bound water molecules form close contacts indicative of OH⋅⋅⋅F hydrogen bonds to the tetrafluoroborate anions that surround the central cavity; two more of these water molecules are located at the two hydrogen‐bond donor sites on the interior surface of the cage associated with the *fac* tris‐chelate sites where there is a convergent array of C−H protons in a region of high positive electrostatic potential.^11^, ^19^

Using an O⋅⋅⋅O or O⋅⋅⋅F distance of ≤3.5 Å as indicative of a hydrogen‐bonding interaction^4^ affords three interactions per water molecule; each one interacts with either three other water molecules, or with two water molecules and a fluoroborate anion. If we also allow a hydrogen‐bonding interaction for each of the two water molecules in the network of CH protons at the electropositive *fac* tris‐chelate sites (Figure 3 b), which collectively act as an H‐bond donor site comparable in strength to phenol,^11^, ^19^ we find that the encapsulated water molecules have, on average, 3.2 hydrogen‐bonding interactions each. Of these, the OH⋅⋅⋅F interactions with tetrafluoroborate are likely to be relatively weak given the poor basicity of tetrafluoroborate compared to water^20^ (HBF_4_ is a stronger acid than H_3_O^+^, and the tetrafluoroborate anion is well known to be a much poorer donor to electropositive metal cations than H_2_O, despite its negative charge). Thus, we can consider 3.2 hydrogen‐bonding interactions per water molecule as an upper limit. Given an average number of hydrogen‐bonding interactions of 3.7 for each molecule in bulk water,^4^ the “energetic frustration” experienced by the set of ten bound water molecules is crudely equivalent to five strong water–water hydrogen bonds, or more if we assume that the OH⋅⋅⋅F interactions are relatively weak.

### NMR studies on guest binding parameters

To dissect the enthalpy and entropy contributions to the hydrophobic effect when guests bind in the cage cavity in water, we have investigated sets of guest pairs which differ by the addition of a methylene group, and for each have measured by NMR spectroscopy the binding constant as a function of temperature, to allow Δ*H* and Δ*S* values to be determined from Van't Hoff plots. A Van't Hoff analysis for an individual guest such as (for example) cycloheptanone, **1**, will provide the overall Δ*H* and Δ*S* contributions to the free energy of binding. On its own this is of limited value as several contributions to binding are conflated: not just the hydrophobic effect but also formation of favourable polar interactions between host and guest, loss of favourable polar interactions of both host and guest with water, van der Waals interactions between host and guest, the entropy penalty associated with combining two species into one species, and so on.10b, 12 However if we compare the Δ*H* and Δ*S* values for binding of cycloheptanone **1** and cyclooctanone **2** and take the difference between them, most of these effects are common to both and therefore cancel out, and we see just the incremental contributions to binding (ΔΔ*H* and *T*ΔΔ*S*) associated with the extra CH_2_ unit.

Previously, we have established that for a range of cyclic ketones from cyclopentanone to cycloundecanone where steric problems on guest binding in **H^w^** did not arise (all guest volumes ≤Rebek's 55 % volume limit),^21^ a linear increase of around 5 kJ mol^−1^ in the free energy of binding for each additional CH_2_ group was observed which is consistent with expectations based on the increased hydrophobic surface area of the guest.^12^ These guests therefore provide an ideal starting point to probe the incremental effects associated with stepwise, predictable increases in the hydrophobic surface area of the guest. Accordingly we have used as guests **1**–**3**, a subset of the set of the cyclic aliphatic ketones,^12^ as well as some cyclic aliphatic lactams **4**–**6** of comparable size, for which (like the ketone series) addition of a single methylene group causes no steric problems for guest binding and results in predictable changes to Δ*G*.

**Scheme 1:**
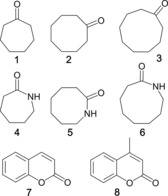
The series of guests used in this work.

**Figure 4:**
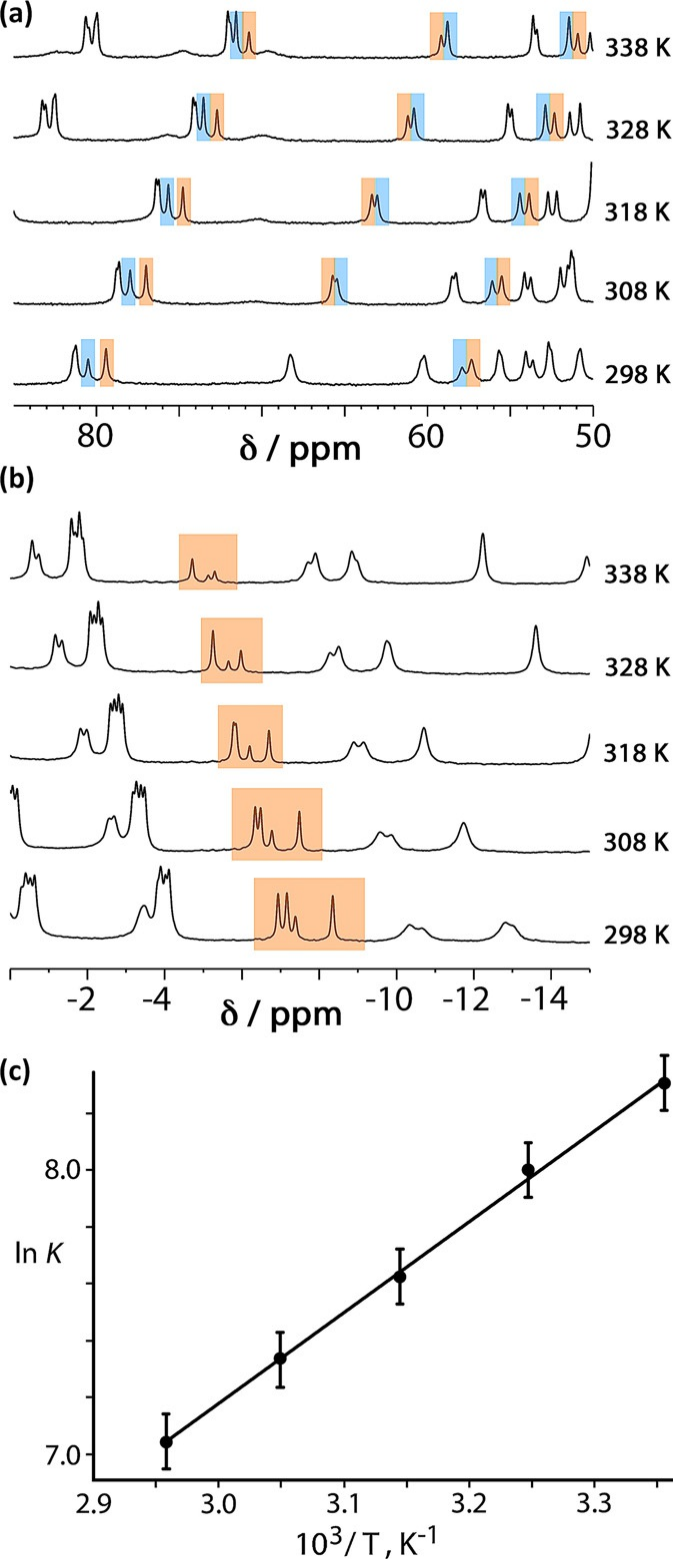
Results of variable‐temperature ^1^H NMR measurements on a mixture of **H^w^** (0.2 mm) and **2** (0.53 mm). a) Evolution of the spectrum in the 50–85 ppm range showing the change in intensity of signals associated with the cage for free **H^w^** (highlighted in blue) and the **H^w^**⋅**2** complex (highlighted in orange); individual integrals could be measured by deconvolution. b) Evolution of the spectrum in the 0 to −14 ppm range showing the variation in amount of bound (and paramagnetically shifted) guest as a function of temperature. c) Van't Hoff plot from which the Δ*H* and Δ*S* data in Table 1 were derived; error bars assume uncertainties of 10 % in binding constant values.

All of the guests evaluated (Scheme 1) bind in the **H^w^** cavity in water in slow exchange on the NMR timescale, such that binding constants can be simply evaluated by integration of separate signals for **H^w^** and **H^w^⋅G** at known total **H^w^** and **G** concentrations. The paramagnetism of the cage complexes greatly facilitates this by dispersing the individual ^1^H resonances over a range of nearly 200 ppm, with the Co^II^ ions acting as excellent shift agents.^10^, ^11^, ^13^, ^22^ This allows similar but slightly different signals for a particular ^1^H environment associated with empty and guest‐containing cages to be distinguished and integrated separately (Figure 4 a). In addition, the paramagnetism means that any bound guest has its ^1^H signals substantially shifted to the region from −4 to −10 ppm, allowing integration of signals for free and bound guest separately (Figure 4 b).

The guests were selected such that their binding constants lie in the range that can be accurately evaluated at typical NMR concentrations, that is, 10^2^–10^4^ m ^−1^. For each **H^w^**/**G** pair the binding constant was measured over several temperatures such that each variable temperature series had at least five data points; changing the temperature further resulted in *K* values becoming too large (at low temperature) or too small (at high temperature) to be able to integrate signals accurately for the purpose of obtaining binding constants. In all cases, the Van't Hoff plots of ln*K* versus 1/*T* afforded straight lines with a positive slope indicating that guest binding is exothermic (Figure 4 c). Δ*G*, Δ*H* and *T*Δ*S* values for each guest binding (at 298 K) are collected in Table 1.

**Table 1:** Thermodynamic parameters for guest binding in **H^w^** in water derived from Van't Hoff plots based on variable‐temperature ^1^H NMR data. Estimated uncertainties are ±1 kJ mol^−1^ in Δ*H* and *T*Δ*S* (see the Supporting Information).

Guest	Δ*G* [kJ mol^−1^] (298 K)	Δ*H* [kJ mol^−1^]	ΔΔ*H* [kJ mol^−1^]	*T*Δ*S* [kJ mol^−1^] (298 K)	*T*(ΔΔ*S*) [kJ mol^−1^] (298 K)
**1**	−15	−22		−7	
			−**5**		**+1**
**2**	−21	−27		−6	
			−**5**		**0**
**3**	−26	−32		−6	
					
					
**4**	−9	−8		+1	
			−**4**		**0**
**5**	−13	−12		+1	
			−**5**		**0**
**6**	−18	−17		+1	
					
					
**7**	−22	−40		−18	
			−**5**		**0**
**8**	−27	−45		−18	

For the cyclic ketones **1**–**3** we can immediately see that, in every case, guest binding is enthalpy‐driven with a large, favourable Δ*H* contribution and a much smaller (and unfavourable) *T*Δ*S* contribution to the binding free energy. This dominance of Δ*H* is consistent with the hydrophobic contribution to guest binding being associated with release of high‐energy water molecules from the cage cavity.^4^, ^5^ However—given the number of different contributions that there might be to Δ*H* and *T*Δ*S*—this is not wholly conclusive. Better evidence comes from examination of the differences between adjacent pairs of guests in the homologous series, from which the ΔΔ*H* and *T*ΔΔ*S* values associated solely with binding of the extra CH_2_ group can be extracted.

Thus, for guest **1** we find that Δ*H* for guest binding is −22 kJ mol^−1^, and that Δ*S* for guest binding is −24 J mol^−1^ K^−1^ which becomes −7 kJ mol^−1^ for *T*Δ*S* at 298 K. For **2** we find that Δ*H* for guest binding is substantially more favourable at −27 kJ mol^−1^ but *T*Δ*S* at 298 K is almost unchanged at −6 kJ mol^−1^. The difference between the pairs of Δ*H* and *T*Δ*S* values (i.e. the values of ΔΔ*H* and *T*ΔΔ*S*) is striking. On addition of one CH_2_ group to the guest skeleton, which results in a free energy improvement to guest binding that can be ascribed to the hydrophobic effect,^12^ the favourable ΔΔ*H* (5(±1) kJ mol^−1^) is much larger than the favourable *T*ΔΔ*S* (1(±1) kJ mol^−1^ at 298 K). A very similar situation arises for the comparison between **2** with **3**: the additional free energy for guest binding is dominated by the enthalpy contribution ΔΔ*H* (5(±1) kJ mol^−1^) with no significant change in *T*ΔΔ*S*.

Within the cyclic ketone series we could not extend the study further, as the smaller and larger members of the series have binding constants outside the window where they can reliably be measured from integration of ^1^H NMR spectra of cage/guest systems in slow exchange. However the cyclic amides **4**–**6** provided two more independent measurements of the same incremental effect (Table 1). The absolute values of −Δ*G* across guest series **4**–**6** are significantly reduced compared to guests **1**–**3** because of the greater hydrophilicity of the lactam guests compared to ketones (replacement of a CH_2_ group by an NH group). Apart from this expected effect however, the value of −Δ*G* for guest binding increased by ca. 5 kJ mol^−1^ per additional methylene group, exactly as we observed with the cyclic ketone series.^12^ Most importantly for this work, the temperature dependent studies of binding demonstrated the same two key points as were observed using the ketone series. These are: 1) for all three guests **4**–**6** the binding free energy is substantially dominated by Δ*H*, with the values of *T*Δ*S* for these guests being close to 0 (i.e., Δ*G* ≈Δ*H* at 298 K); and 2) comparisons between **4**/**5** and **5**/**6** reveal incremental changes associated with each extra methylene group that are mostly enthalpy‐based (ΔΔ*H* 4–5 kJ mol^−1^) with the *T*ΔΔ*S* increment being insignificant (within experimental uncertainty).

Finally, to see if this consistent incremental effect extended to structurally somewhat different guests, we also compared the binding parameters of coumarin (**7**) and 4‐methylcoumarin (**8**). Exactly the same general behaviour was observed, with the absolute values of Δ*H* being substantially larger than those of *T*Δ*S* at 298 K for both guests, and the incremental changes associated with the additional CH_2_ group in **8** over **7** being about 5 kJ mol^−1^ for ΔΔ*H* and about 0 kJ mol^−1^ for *T*ΔΔ*S*.

The agreements in ΔΔ*H* and *T*ΔΔ*S* values per CH_2_ group, based on five independent pairwise comparisons across three guest series, is remarkably good and paints a very clear picture of an enthalpy‐dominated hydrophobic effect. This is entirely consistent with the “high‐energy water” model of the hydrophobic effect that applies to small, concave surfaces which surround cavities containing a few water molecules in an arrangement that frustrates optimal hydrogen‐bonding.^3^, ^4^, ^5^, ^6^ It is also consistent with the crystal structure of the hydrated cage which showed a clear degree of hydrogen‐bond frustration in the bound water cluster. Note that the consistency of the ΔΔ*H* values implies that the degree of frustration of the extra water released by each incremental addition of a CH_2_ group is the same, which is something that need not necessarily be true. We can imagine that displacing the first water molecule from a cluster of ten, and transferring it to the bulk solution, results in a change in hydrogen‐bonding that is not the same as would arise from displacement of the fifth, or tenth, water molecule. As the guests in Scheme 1 fall within a fairly narrow size range (between 31 and 43 % of the cavity volume),^11^, ^12^ it is reasonable that the incremental effect of the extra methylene group in each pairwise comparison is similar in each case.

Some of the highest Δ*H* values for guest binding known in synthetic hosts, associated with binding of guests such as adamantanes and ferrocene derivatives in CB7, are in the range 60–90 kJ mol^−1^.^4^ Our coefficient of 5 kJ mol^−1^ per CH_2_ group^12^ is close to this, affording favourable enthalpy contributions to binding of up to 50 kJ mol^−1^ for the hydrocarbon component of cycloundecanone, for example, which optimally fills the cavity;^12^ this Δ*H* value is of a magnitude consistent with formation of an additional five hydrogen bonds when all ten water molecules are liberated by this guest.

Further, it is not just the magnitude of the enthalpy contribution to hydrophobic binding that is significant, but the extent to which this dwarfs the entropy component—a characteristic signature of strong binding associated with liberation of high‐energy water from cavities.^4^ We note that the volume of the cavity of **H** and **H^w^**, at around 400 Å^3^, is about 10 % larger than the cavity of CB8, and the number of bound water molecules (10) lies between what occurs in CB7 and CB8 (8 or 12, respectively);^5^, 7a so **H^w^** should lie somewhere between CB7 and CB8 in its capability to bind high‐energy water in the cavity, in agreement with the dominance of the enthalpy effect in our results.

## Conclusions

In conclusion, a combination of crystallographic and NMR spectroscopic studies has been used as a basis for analysing enthalpy and entropy contributions to the hydrophobic contribution to guest binding in a small M_8_L_12_ cubic cage host. By comparing the binding properties of five pairs of similar guests that differ only by a methylene group we could filter out the other thermodynamic contributions to binding of each individual guest, and show that each additional hydrophobic methylene group in a guest resulted in a remarkably consistent additional enthalpy contribution of around 5 kJ mol^−1^, with the incremental entropy contribution being much less significant. The hydrophobic contribution to guest binding is therefore dominated by enthalpy, and the environment we observe around the bound water cluster in the crystal structure provides an explanation for this. This work therefore demonstrates important principles which can be exploited in the design of new hosts for binding guests strongly in water, a key goal in supramolecular chemistry.

## Experimental Section

### General details

The cages **H** 10a and **H^w^** ^[10b]^ were prepared as previously described. The guests **1**–**8** were obtained from Sigma–Aldrich and used as received. Binding constants of guests (from which the Δ*G* values in Table 1 were derived) were measured by NMR spectroscopy in D_2_O using a Bruker AV3‐400 spectrometer as reported in previous papers;^11^, ^12^, ^13^, ^14^, ^15^ temperature dependent NMR measurements used to generate the Van't Hoff plots were performed on the same instrument. All measurements were repeated several times to check for consistency. One illustrative Van't Hoff is in Figure 4, for guest **2**; all others are in the Supporting Information.

### X‐ray crystallography

Crystals of **H**⋅(H_2_O)_28_ were prepared simply by immersing pre‐formed crystals of **H** as its MeOH solvate10a in water for a few hours. Crystallographic data for **H**⋅(H_2_O)_28_: C_336_H_272_B_16_Co_8_F_64_N_72_O_28_, *M*=7626.64 g mol^−1^, monoclinic, space group *C*2/*c*, *a=*32.9721(4), *b=*29.9227(5), *c=*40.0392(6) Å, *β*=96.2151(12)°, *U*=39 271.0(10) Å^3^, *Z=*4, *ρ*_calcd_=1.290 g cm^−3^, *T=*100(2) K, *λ*=0.71073 Å, *μ*=0.426 mm^−1^. 109 356 reflections with 2*θ*_max_=50° were merged to give 34 370 independent reflections (*R*_int_ *=*0.0448). Final *R*_1_ [for data with *I*>2*σ*(*I*)]=0.110; w*R*_2_ (all data)=0.368. The data collection was performed by the EPSRC National Crystallography Service at the University of Southampton.^23^ Data were corrected for absorption using empirical methods (SADABS)^24^ based upon symmetry‐equivalent reflections combined with measurements at different azimuthal angles. The structure was solved and refined using the SHELX suite of programs.^25^ The asymmetric unit contains one half of the molecule which lies astride an inversion centre. As usual with structures of this family, disorder of anions/solvent molecules resulted in weak scattering, necessitating use of extensive geometric and displacement restraints to keep the refinement stable. The presence of large regions of diffuse electron density which could not be modelled required use of the SQUEEZE function in PLATON. Full details are in the CIF. The cluster of ten water molecules in the cage cavity is well‐behaved, being disordered over two similar positions with site occupancies of 0.55 and 0.45, as are some of the fluoroborate anions which interact with the water cluster; see main text and the Supporting Information.

## Conflict of interest

The authors declare no conflict of interest.

## Supporting information

As a service to our authors and readers, this journal provides supporting information supplied by the authors. Such materials are peer reviewed and may be re‐organized for online delivery, but are not copy‐edited or typeset. Technical support issues arising from supporting information (other than missing files) should be addressed to the authors.

Supplementary
