# Supplementary material for: Binding of Hydrophobic Guests in a Coordination Cage Cavity is Driven by Liberation of “High‐Energy” Water
Source: Chemistry. 2017 Nov 30;24(7):1554–60. doi: 10.1002/chem.201704163 (PMC5814853; doi:10.1002/chem.201704163)
Supplement: Supplementary file 1 — Supplementary [file CHEM-24-1554-s001.pdf]

CHEMISTRY
A European Journal

Supporting Information

Binding of Hydrophobic Guests in a Coordination Cage Cavity is Driven by Liberation of "High-Energy" Water

Alexander J. Metherell,[a] William Cullen,[a] Nicholas H. Williams,[a] and Michael D. Ward*[a, b]

chem_201704163_sm_miscellaneous_information.pdf

Electronic supporting information

Measurements of binding constants (experimental details) p1
Fig. S1/S2 (NMR spectra and Van't Hoff plot for guest **1**) p2
Fig. S3/S4 (NMR spectra and Van't Hoff plot for guest **3**) p3
Fig. S5/S6 (NMR spectra and Van't Hoff plot for guest **4**) p4
Fig. S7/S8 (NMR spectra and Van't Hoff plot for guest **5**) p5
Fig. S9/S10 (NMR spectra and Van't Hoff plot for guest **6**) p6
Fig. S11/S12 (NMR spectra and Van't Hoff plot for guest **7**) p7
Fig. S13/S14 (NMR spectra and Van't Hoff plot for guest **8**) p8
Fig. S15/S16 (Additional pictures of crystal structure) p9
Table S1 (O•••O and O•••F distances associated with guest water molecules) p10

Measurements of binding constants

Binding constants of guests were measured by NMR spectroscopy in D_2O using a Bruker AV3-400 spectrometer. Concentration of host cage $\mathbf{H^w}$ was always 0.2 mM; guest binding was always in slow exchange such that separate signals for free $\mathbf{H^w}$ and the $\mathbf{H^w}$•**G** complex could be observed which could be separately integrated to allow calculation of the binding constant at a range of temperatures. In addition, signals for bound guests appeared in the region −5 to −10 ppm (shifted by the cage paramagnetism) and integration of these compared to those for unbound **G** provided additional measurements of binding constants. When signals for free $\mathbf{H^w}$ and the $\mathbf{H^w}$•**G** complex overlapped they were separated by deconvolution in the Bruker Topspin® software. When this was required the region around overlapping signals was carefully baselined first and then the peaks deconvoluted using a mixed Lorentzian / Gaussian model, manually adjusting parameter such as threshold and peak overlap to ensure a good fit.

This comparison of integral measurements was repeated multiple times across a spectrum whenever distinct signals for free $\mathbf{H^w}$ and the $\mathbf{H^w}$•**G** complex (whether overlapping or completely separate) could be identified, and these were averaged. In addition each measurement was repeated at least twice. The averaging of multiple independent measurements of each cage/guest system at each temperature ensured that issues associated with integral measurements of weak, broad or overlapping signals are minimised, with uncertainties on individual K values in the range 1 – 10%. Thus, for the data analyses shown below, we have allowed a pessimistic uncertainty of ±10% in *all* data points; this gives errors in ΔG of < 1 kJ/mol.

The following figures showing how the ^{1}H NMR spectra changed with temperature for each $\mathbf{H^w}$/**G** pair, followed by the Van't Hoff plot for each system from which the values of ΔH and $T\Delta S$ for binding for each guest (used in Table 1) were derived. Error bars are based on an estimated uncertainty of ±10% in each K value as described above.

Hʷ + guest **1** (cycloheptanone)

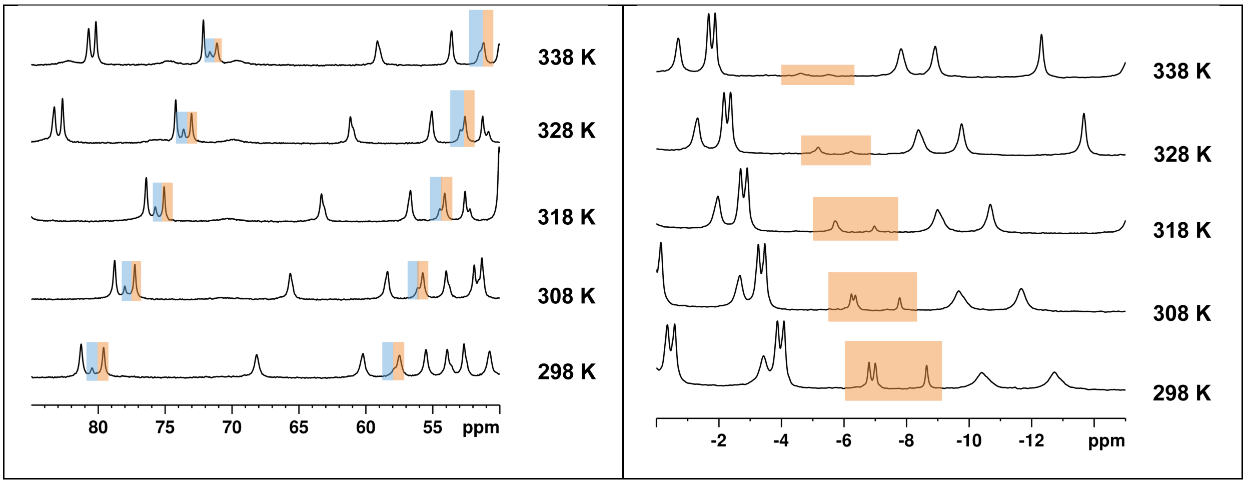

Fig. S1. Parts of the 1H NMR spectrum of a mixture of **H**ʷ (0.2 mM) and guest **1** (8.47 mM) in D_2O at various temperatures. Left: changes in signals of the host when guest binds (blue = free **H**ʷ; red = **H**ʷ•**1** complex). Right: changes in signals for bound guest (highlighted in red).

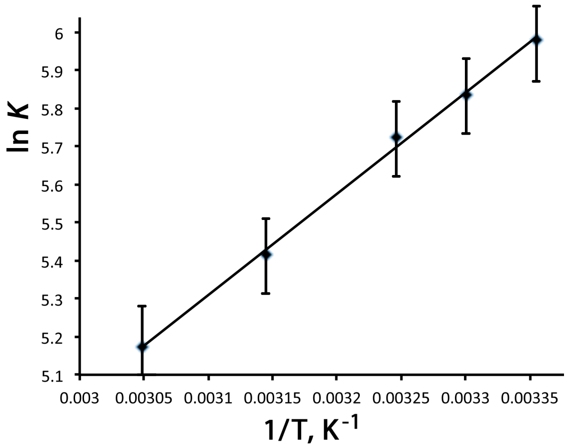

Fig. S2. Van't Hoff plot for the **H**ʷ / **1** system based on the NMR data shown above, from which the data in Table 1 were derived. Error bars are based on an estimated uncertainty of ±10% in each *K* value.

H^w + guest **3** (cyclononanone)

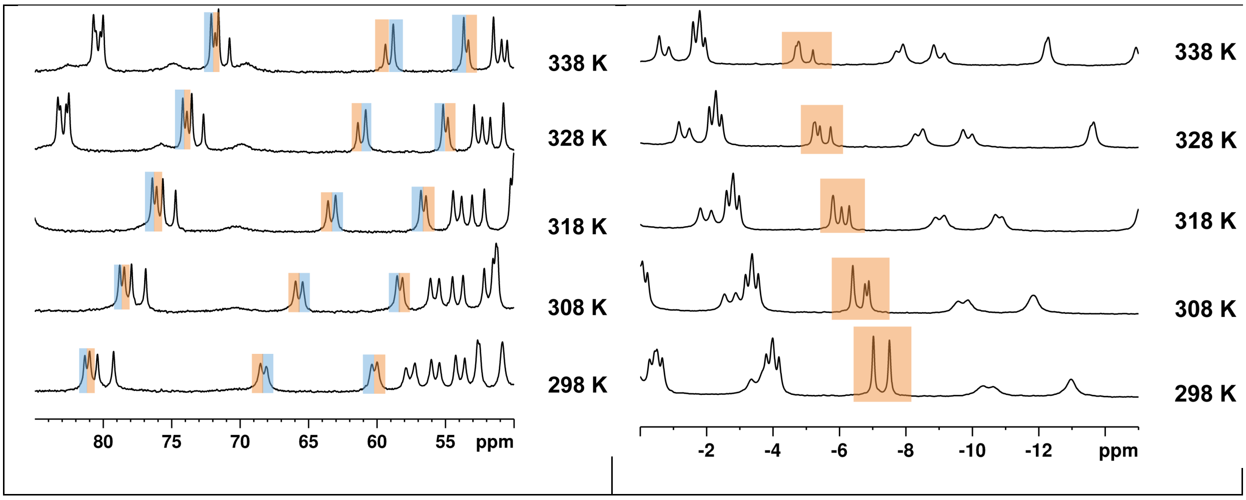

Fig. S3. Parts of the 1H NMR spectrum of a mixture of **H^w** (0.2 mM) and guest **3** (0.14 mM) in D_2O at various temperatures. Left: changes in signals of the host when guest binds (blue = free **H^w**; red = **H^w•3** complex). Right: changes in signals for bound guest (highlighted in red).

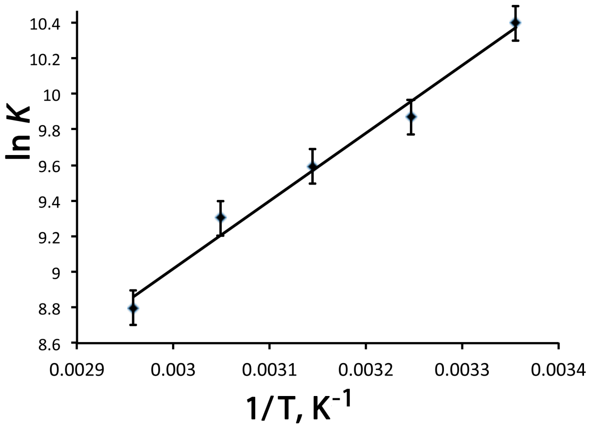

Fig. S4. Van't Hoff plot for the **H^w** / **3** system based on the NMR data shown above, from which the data in Table 1 were derived. Error bars are based on an estimated uncertainty of ±10% in each *K* value.

H^w + guest **4** (caprolactam)

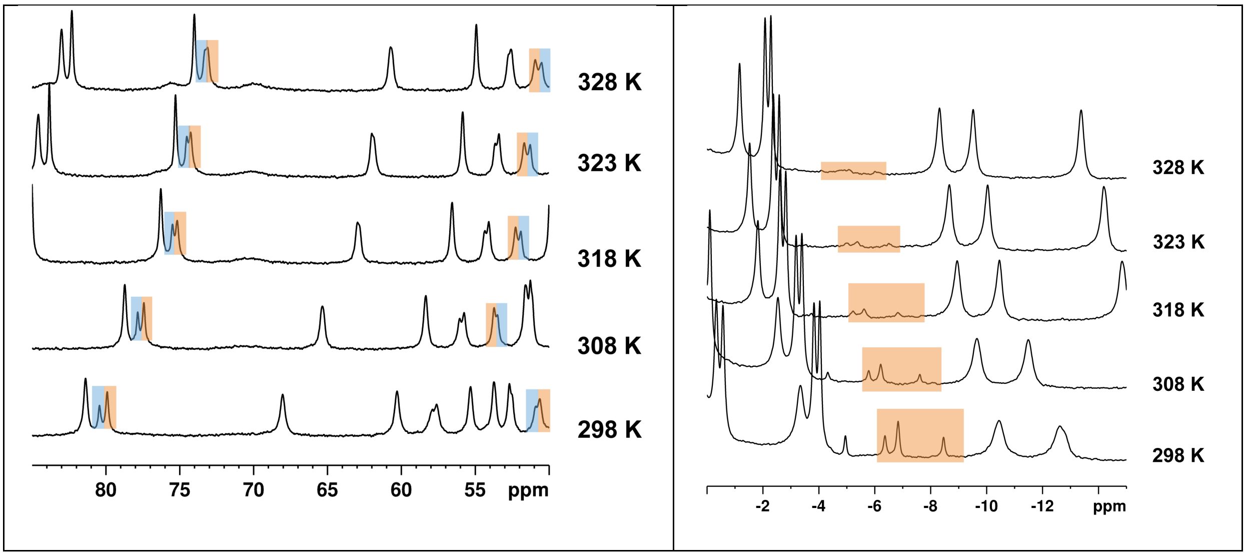

Fig. S5. Parts of the 1H NMR spectrum of a mixture of **H^w** (0.2 mM) and guest **4** (50.1 mM) in D_2O at various temperatures. Left: changes in signals of the host when guest binds (blue = free **H^w**; red = **H^w•4** complex). Right: changes in signals for bound guest (highlighted in red).

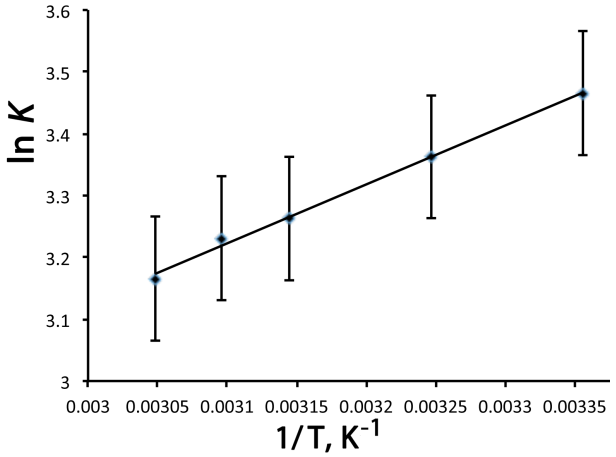

Fig. S6. Van't Hoff plot for the **H^w** / **4** system based on the NMR data shown above, from which the data in Table 1 were derived. Error bars are based on an estimated uncertainty of ±10% in each *K* value.

H^{w} + guest **5** (1-aza-2-cyclooctanone)

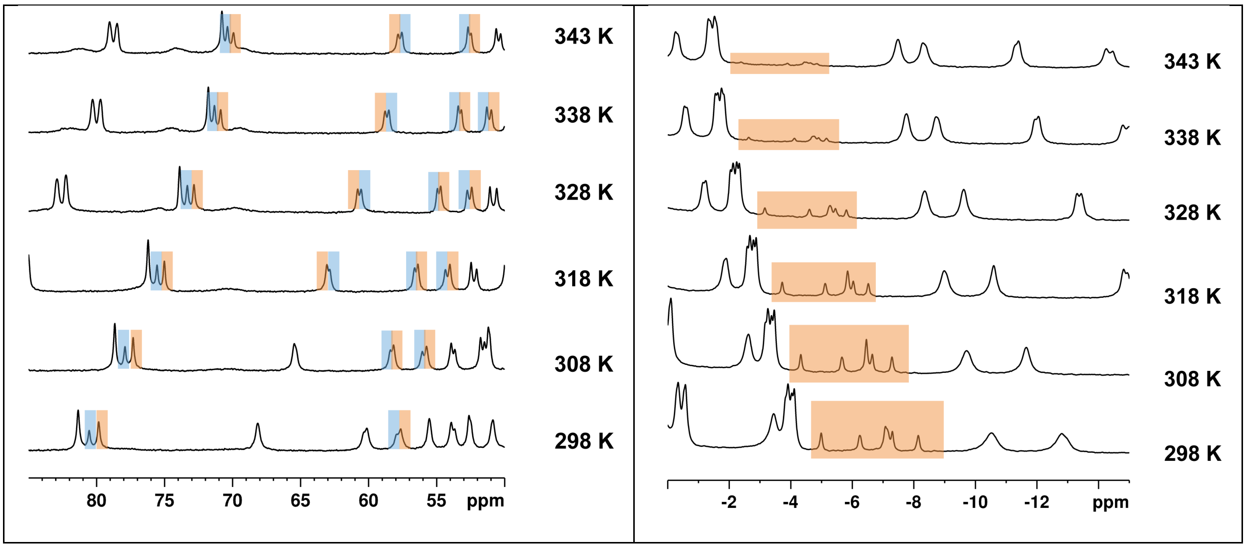

Fig. S7. Parts of the 1H NMR spectrum of a mixture of **H**^{w} (0.2 mM) and guest **5** (9.15 mM) in D_2O at various temperatures. Left: changes in signals of the host when guest binds (blue = free **H**^{w}; red = **H**^{w}**•5** complex). Right: changes in signals for bound guest (highlighted in red).

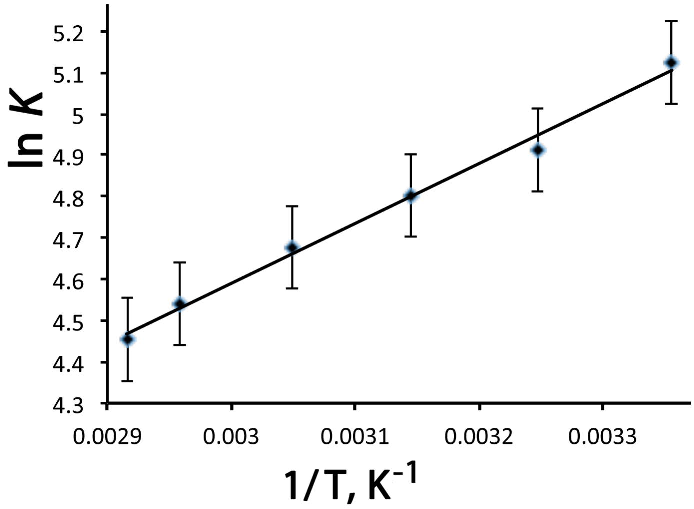

Fig. S8. Van't Hoff plot for the **H**^{w} / **5** system based on the NMR data shown above, from which the data in Table 1 were derived. Error bars are based on an estimated uncertainty of ±10% in each *K* value.

Hʷ + guest **6** (1-aza-2-cyclononanone / 8-octanolactam)

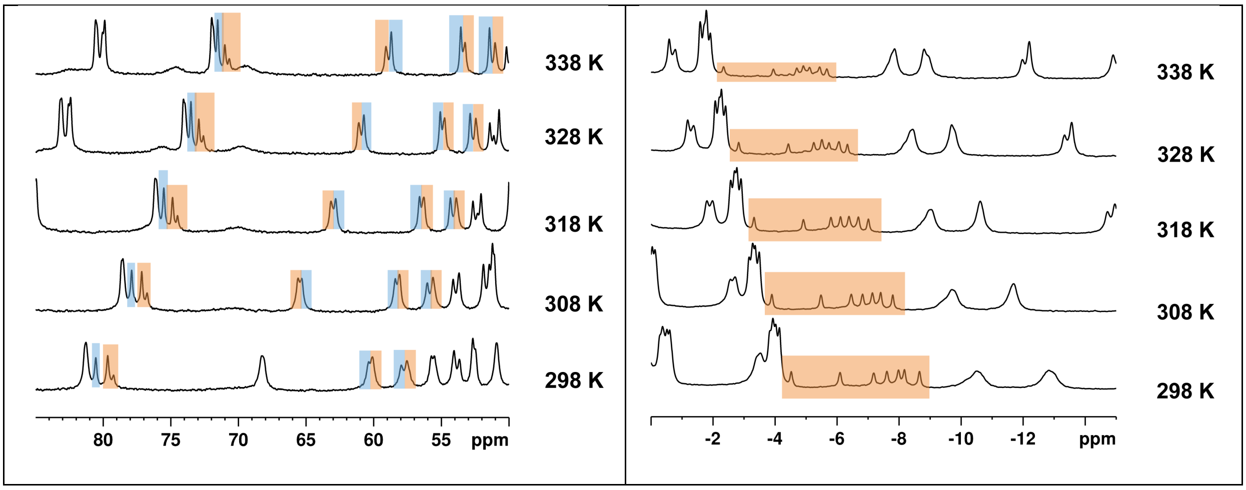

Fig. S9. Parts of the 1H NMR spectrum of a mixture of $\mathbf{H^w}$ (0.2 mM) and guest **6** (1.33 mM) in D_2O at various temperatures. Left: changes in signals of the host when guest binds (blue = free $\mathbf{H^w}$; red = $\mathbf{H^w}$•**6** complex). Right: changes in signals for bound guest (highlighted in red).

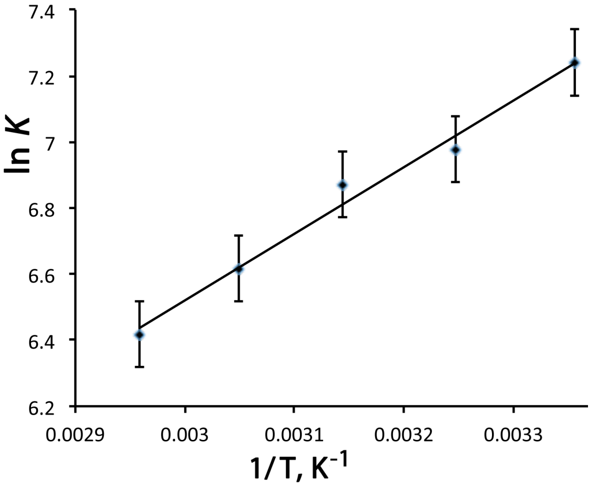

Fig. S10. Van't Hoff plot for the $\mathbf{H^w}$ / **6** system based on the NMR data shown above, from which the data in Table 1 were derived. Error bars are based on an estimated uncertainty of ±10% in each *K* value.

H^{w} + guest **7** (coumarin)

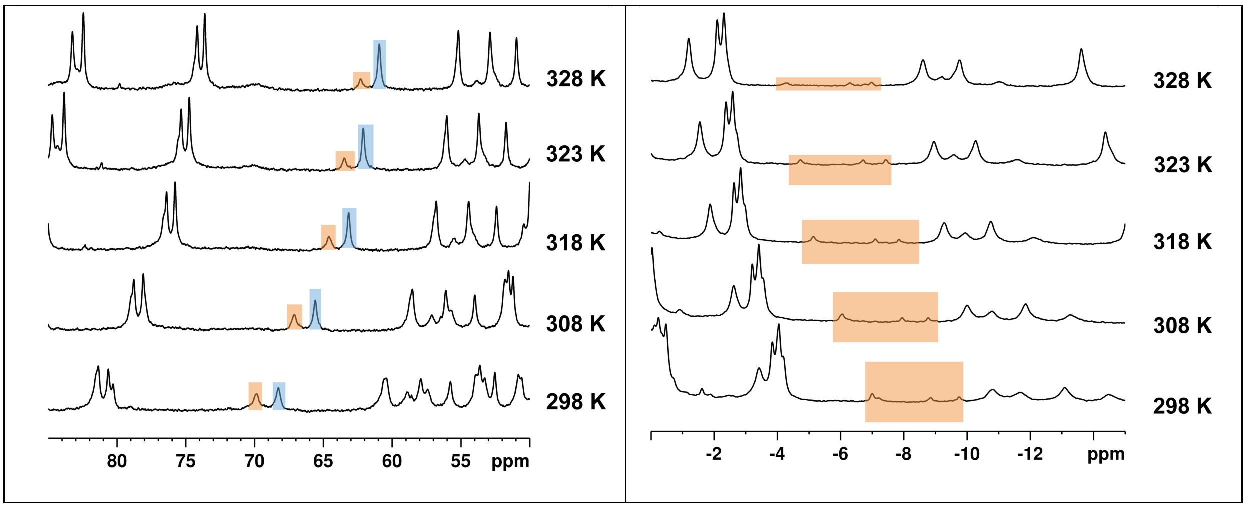

Fig. S11. Parts of the 1H NMR spectrum of a mixture of **H**^{w} (0.2 mM) and guest **7** (0.25 mM) in D_2O at various temperatures. Left: changes in signals of the host when guest binds (blue = free **H**^{w}; red = **H**^{w}•**7** complex). Right: changes in signals for bound guest (highlighted in red).

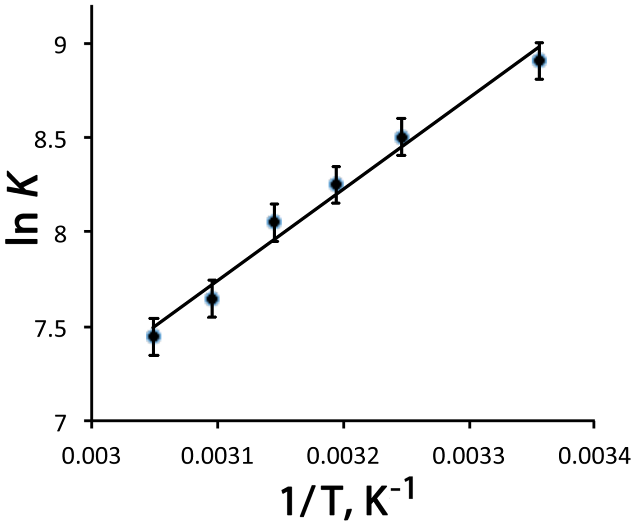

Fig. S12. Van't Hoff plot for the **H**^{w} / **7** system based on the NMR data shown above, from which the data in Table 1 were derived. Error bars are based on an estimated uncertainty of ±10% in each *K* value.

H[w] + guest **8** (4-methyl-coumarin)

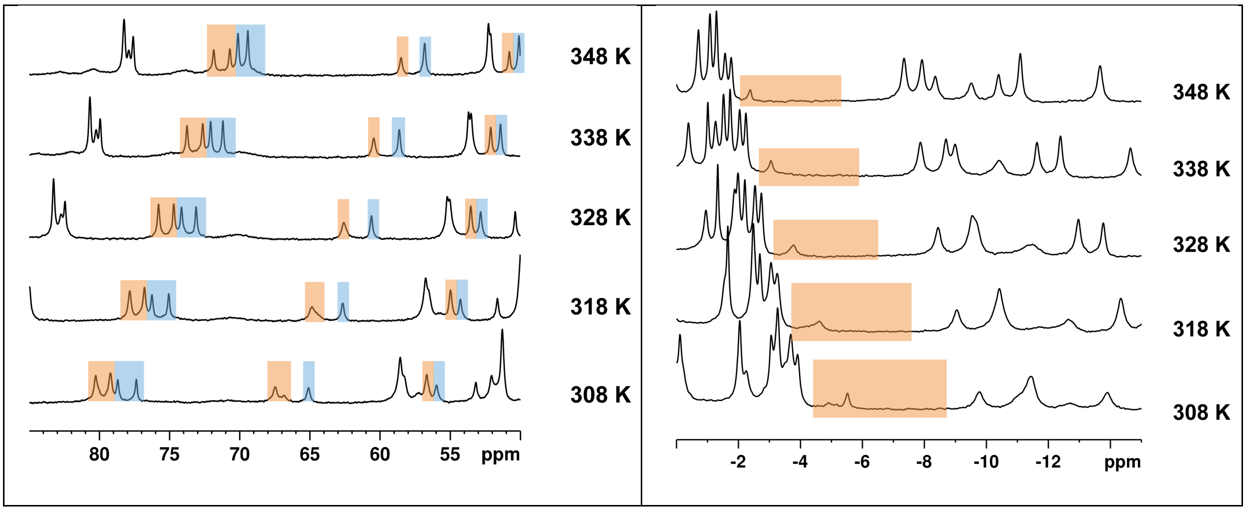

Fig. S13. Parts of the 1H NMR spectrum of a mixture of **H**[w] (0.2 mM) and guest **8** (0.25 mM) in D_2O at various temperatures. Left: changes in signals of the host when guest binds (blue = free **H**[w]; red = **H**[w]**•8** complex). Right: changes in signals for bound guest (highlighted in red).

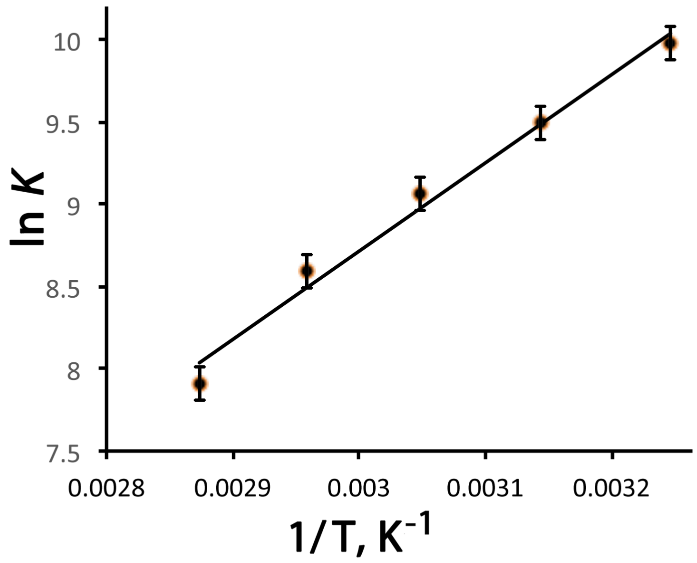

Fig. S14. Van't Hoff plot for the **H**[w] / **8** system based on the NMR data shown above, from which the data in Table 1 were derived. Error bars are based on an estimated uncertainty of ±10% in each *K* value.

Additional X-ray crystallographic figures

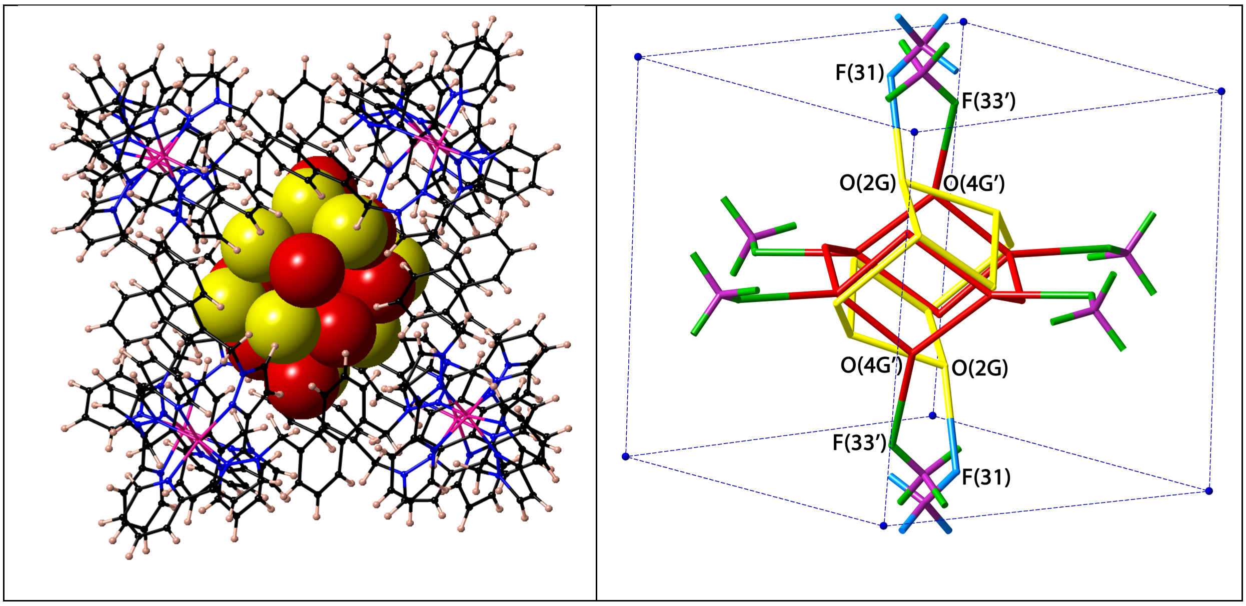

Fig. S15. Superposition of the two disorder components of the set of bound water molecules in the cage cavity (red = major component, yellow = minor component). (Left) a view of the complete cage with the guest O atoms shown space-fillling; (right) a view showing how disorder of bound water molecules is associated with disorder in the positions of two of the fluoroborate anions in the cage portals (containing F31 / F33).

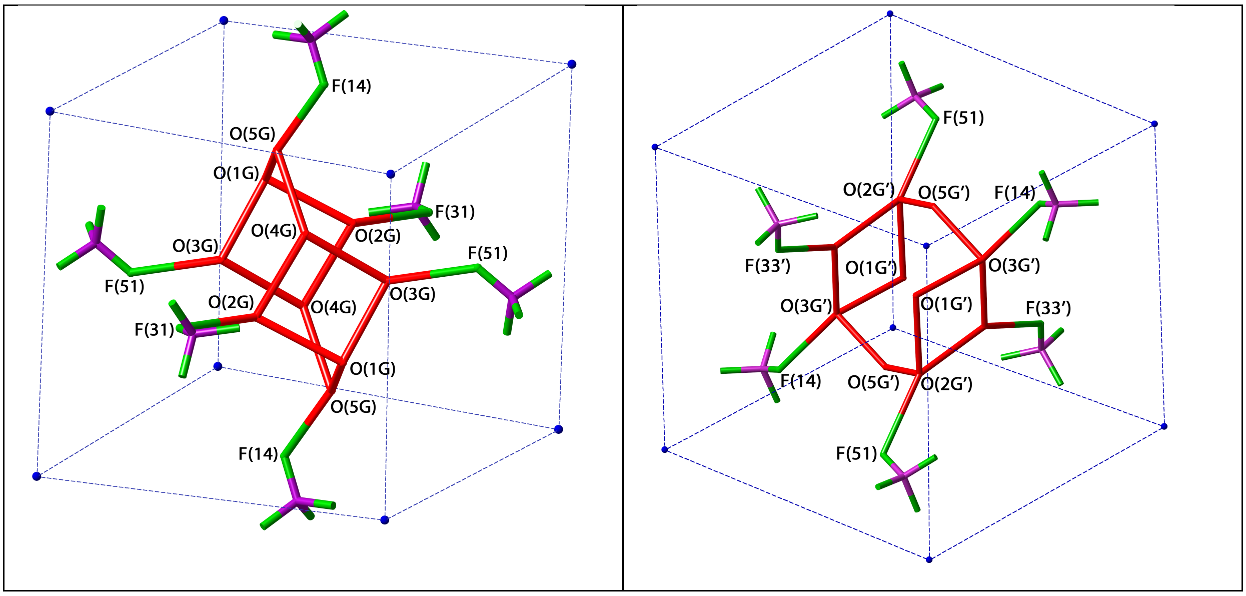

Fig. S16. Views showing in wireframe the arrangement of water molecule guests in the cavity and their interactions with the fluoroborate anions in the portals (left, major component; right, minor component).

Table S1: O•••O and O•••F distances associated with guest water molecules

MAJOR DISORDER COMPONENT		MINOR DISORDER COMPONENT	
O(5G) – O(1G)	2.96 Å	O(3G') – O(5G')	2.83 Å
O(5G) – O(4G)	2.81	O(3G') – O(1G')	2.62
O(1G) – O(2G)	2.92	O(3G') – O(4G')	2.74
O(1G) – O(3G)	2.76	O(2G') – O(5G')	2.74
O(2G) – O(4G)	2.92	O(2G') – O(4G')	2.91
O(3G) – O(4G)	3.05	O(1G') – O(2G')	2.89
O(5G) – F(14)	2.77	O(3G') – F(14)	2.95
O(2G) – F(31)	3.02	O(2G') – F(51)	2.90
O(3G) – F(51)	3.20	O(4G') – F(33')	3.08

Average O-O: 2.90 Å
Average O-F: 3.00 Å

Average O-O: 2.79 Å
Average O-F: 2.98 Å
